# Dynamic Indicators of Adherence and Retention in Adults Using a Digital Mental Health App: Longitudinal Observational Analysis From the Brighten Study

**DOI:** 10.2196/69464

**Published:** 2025-12-22

**Authors:** Dylan Hamitouche, Youcef Barkat, Deven Parekh, Eva Hammer, David Benrimoh

**Affiliations:** 1 Department of Medicine, McGill University, Montreal, QC, Canada; 2 Douglas Mental Health University Institute, 6875 LaSalle Boulevard, Montreal, QC, H4H 1R3, Canada, 1 514-239-0428; 3 Department of Biochemistry, Université de Montréal, Montreal, QC, Canada; 4 Department of Psychology, McGill University, Montreal, QC, Canada; 5 Department of Psychiatry, McGill University, Montreal, QC, Canada

**Keywords:** mHealth, digital health, mobile health, digital biomarkers, adherence, depression, smartphone, digital interventions

## Abstract

**Background:**

Making optimal use of mobile health technologies requires the validation of digital biomarkers, which in turn demands high levels of participant adherence and retention. However, current remote digital health studies have high attrition rates and low participant adherence, which may introduce bias and limit the generalizability of their findings.

**Objective:**

This study aimed to identify longitudinal indicators of participant retention and adherence, which may serve to develop strategies to improve data collection in digital health studies and improve understanding of how study cohorts are shaped by participant withdrawal and nonadherence.

**Methods:**

We performed secondary analyses on the Brighten study, which consisted of 2 remote, smartphone-based randomized controlled trials evaluating mobile apps for depression treatment, enrolling 2193 participants in total. Participants were asked, after baseline assessment, to complete 7 digital questionnaires regularly. We assessed adherence to digital questionnaires, engagement (postbaseline participation), and retention rates (the proportion of participants who continued completing questionnaires over time) as outcomes. We investigated the relationship between these outcomes and both static measures (eg, demographics and average questionnaire scores) and dynamic measures (eg, changes in questionnaire scores over time).

**Results:**

The study included 2201 participants, of whom 1093 completed at least 1 nonbaseline questionnaire, with a median completion rate of 37.6% (IQR 15.5%-67.9%). We found significantly higher adherence rates in participants who were less depressed on average over the course of the study (*t*_752_=−5.63; *P*<.001) and in those who perceived clinical improvement (*t*_744_=3.78; *P*=.001). There were significant demographic differences in adherence and engagement, including differences by gender, race, education, income, and income satisfaction. Participants who were more depressed at baseline were more likely to withdraw before completing any nonbaseline questionnaire (*t*_1917_=−2.53; *P*=.01). However, participants who showed improvement in depressive symptoms during the study showed better adherence (Mann-Whitney U=127,084; *P*<.001) and retention (hazard ratio 0.78, 95% CI 0.67-0.91; *P*=.002), despite showing greater depressive symptoms at baseline.

**Conclusions:**

We show that participants’ clinical trajectory of depressive symptoms, as well as their perception of improvement, are important indicators of engagement, adherence, and retention. Expanding knowledge regarding these longitudinal indicators may improve interpretation of outcomes and help build strategies to improve retention and adherence in future clinical trials.

## Introduction

Over the past decade, researchers have dedicated significant effort to identifying clinically useful biomarkers for preventing, diagnosing, and treating psychiatric disorders. Digital biomarkers have garnered considerable attention, as they enable the observation of long-term patterns and trends outside of the hospital or clinic environment, potentially enhancing our understanding of the course of mental illnesses [1,2]. Identifying reliable digital biomarkers has the potential to significantly improve the accuracy of diagnoses, predictions of clinical outcomes (eg, suicidal ideation [3]), and treatment decision-making regarding psychiatric disorders [4-6]. Furthermore, collecting digital biomarkers is cost-effective [7,8] and allows the possibility to provide patients with feedback through electronic reports, giving them ownership of their own medical data [9-12].

One major challenge for the effectiveness of mobile health technologies is the identification and validation of digital biomarkers [13-16]. This process requires high levels of participant adherence and retention to ensure sufficient data are collected for validation purposes. Active data collection methods, such as digital questionnaires, which are often needed to help validate or contextualize digital biomarkers, intensify this challenge as they demand more time and effort than passive data collection methods such as actigraphy [17,18]. While passive measures may be useful, their validation depends on access to actively collected assessments of patient status. Therefore, understanding the drivers of participant adherence to data collection methods requiring active participation is crucial for the effective design and validation of digital biomarker collection platforms. Eysenbach described the “law of attrition,” the tendency for a significant proportion of users to drop out or stop using eHealth mobile apps before completing a trial [19-23]. This dropout may shape the cohort over time in a way that does not represent the initially recruited study population in terms of demographic features, symptomatology, and clinical outcomes, therefore inducing bias in the analyses [16,24].

In studies requiring active remote digital participation, such as completing questionnaires, a few static parameters (ie, those that do not change over the course of the study) have been associated with adherence rates (the degree to which participants follow the study protocol and complete assessments), engagement rates (the proportion of participants who engage in the study after baseline, ie, who complete at least 1 nonbaseline questionnaire), and retention rates (the proportion of participants who continue to complete questionnaires over time). Previous studies suggest that participants who are more depressed at baseline, younger, and less educated tend to have lower assessment adherence rates [16,25,26]. Retention rates can also vary between treatment arms involving mobile apps versus control groups, where control groups with lower engagement requirements may often show better retention, and between ethnic groups [27]. A lower annual income also tends to be significantly associated with poorer study engagement [26].

These static parameters, often of a demographic or symptomatologic nature and considered as averages rather than longitudinal measures, provide meaningful insight into the question of participant engagement in active digital phenotyping. However, there has been less exploration of how dynamic parameters, such as longitudinal symptom change, might affect engagement. Therefore, the objective of this study was to identify novel predictive longitudinal markers of adherence, engagement, and retention to regular self-rated assessments in participants enrolling in a remote digital health study requiring active participation. To do so, we conducted secondary analyses on the Brighten study, a large longitudinal digital intervention study.

## Methods

### Study Design

The Brighten study consists of 2 completely remote, smartphone-based randomized controlled trials assessing the efficiency of mobile apps for treating depression. The study recruited 2193 participants from the United States through online advertisements, with recruitment for the first version (V1) beginning in August 2014 (recruitment lasted 5 months) and the second version (V2) starting in August 2016 (recruitment lasted 7 months), enrolling 1110 and 1083 participants, respectively [25,26]. Participants were included in the Brighten study if they had a score of 5 or higher on the Patient Health Questionnaire (PHQ)-9 or a score of 2 or greater on PHQ item 10 [28]. The study evaluated 3 interventions over 12 weeks: (1) iProblemSolve (iPST), an app designed by UCSF for problem-solving therapy [25,29,30]; (2) Project: EVO (Akili Interactive Labs), a therapeutic video game; and (3) Health Tips, an app offering strategies to improve mood [28]. The group using Health Tips was considered the control group. In those 2 randomized controlled trials, various questionnaires were sent to participants via smartphone notifications. Most assessments were submitted daily, weekly, or biweekly, but some questionnaires were only sent at baseline. The Brighten study also collected passive digital communication data from the V1 and V2 cohorts, along with passive mobility features from the V2 cohort. While V2 followed a protocol very similar to V1 (same interventions and active data collection methods), it specifically aimed to increase participation among individuals of Hispanic or Latino ethnic backgrounds to assess the feasibility of using digital mental health tools in this population [28].

### Intervention Apps

The iPST app implements a 7-step problem-solving therapy model to manage mood: participants select a goal and are guided through a structured action plan. Participants assigned to this app were instructed to use it at least once per week, consistent with typical clinical use [31].

Participants assigned to Project: EVO were encouraged to use it 6 times per week for approximately 30 minutes per day. This video game–based app aimed at enhancing cognitive skills related to depression using adaptive algorithms to adjust difficulty to the user’s proficiency [32]. Previous studies of the app’s predecessor showed that this dosage improved cognitive control and depressive symptoms in older adults [32,33].

The control group used an app providing daily health tips, such as self-care (eg, showering) or physical activity (eg, walking). Participants in this group were not required to act on the tips, serving as a supportive control condition.

Importantly, all participants, regardless of their assigned intervention, completed their self-rated questionnaires in a separate study app. Our analyses specifically focused on adherence, engagement, and retention with these assessments.

### Measures

Demographics such as gender, education level, working status, income satisfaction, last-year income, marital status, race, and age, as well as parameters regarding study involvement (how they heard about the study, device used, and study version), are provided in Multimedia Appendix 1. These parameters, along with baseline PHQ-9 results, were imputed using MissForest, a nonparametric missing value imputation for mixed-type data, if less than 30% of the data were missing for that specific metric. This threshold was chosen based on validation studies demonstrating the reliability of MissForest under these conditions [34]. Participants were sent 7 questionnaires to complete at a predefined frequency specific to each questionnaire: PHQ-9 [35] (weekly for the first 4 weeks and then biweekly) and PHQ-2 [36] (daily); a 3-item sleep assessment assessing sleep onset latency, sleep duration, and time awake at night (weekly); the Sheehan Disability Scale (SDS) [37] (weekly for the first 4 weeks and then biweekly); the Patients Global Impression of Change (PGIC) Scale [38] (weekly); a survey inquiring about the use of mental health services (weekly); a survey inquiring about study app satisfaction (deployed at weeks 4, 8, and 12); and a survey inquiring about the use of other health-related apps (deployed at weeks 1, 4, 8, and 12) [28]. All questionnaires included in our analyses are provided in Multimedia Appendix 1.

### Outcomes

We evaluated 3 key outcomes: adherence, engagement, and retention. To evaluate individual adherence, each participant’s average completion rate was calculated as the ratio of completed questionnaires to the total number they were expected to complete. Engagement was binary: participants were considered engaged if they completed at least one nonbaseline assessment and disengaged otherwise. Therefore, disengaged participants, by definition, had a completion rate of 0%. Engaged participants were further divided into high- and low-completion groups, based on the median average completion rate. Based on previous research, engaged participants were also categorized into “improvers” and “nonimprovers” based on the difference between their baseline PHQ-9 score and their latest PHQ-9 score, with a minimum clinically important difference set at 5 points [39]. Therefore, engagement captures a binary distinction (completion beyond baseline), adherence represents a continuous measure of completion, and the subgrouping serves only as a descriptive stratification of engaged participants. Participant retention was determined as the duration for which participants continued completing a specific representative questionnaire (eg, PHQ-9) before they withdrew or stopped responding. It is important to consider that participants in the iPST and Project: EVO intervention groups were asked to use the apps as part of their treatment, but data on their engagement or adherence to the interventions were not available, so treatment adherence was not included as an outcome.

### Statistical Methods

To compare engagement and average completion rates across demographic groups, Mann-Whitney U tests were used for 2-group comparisons, and Kruskal-Wallis tests followed by Dunn post hoc tests were applied for comparisons involving more than 2 groups.

A *t* test was conducted on average questionnaire scores and baseline scores between high and low completion groups, evaluating the importance of symptom severity as a static indicator of adherence. A *t* test was also conducted on baseline PHQ-9 scores between the engaged and disengaged groups to assess whether engagement was associated with baseline depressive symptoms.

To assess the potential of symptomatology as a dynamic predictor of adherence, trends in questionnaire scores over time between high and low completion groups were investigated using a repeated measures generalized linear model (GLM).

Elastic net regression with Shapley additive explanations (SHAP) values [40] was used to identify the top 5 predictive features for average completion rate, engagement status (engaged/disengaged), and completion group (high/low), incorporating demographic parameters, average questionnaire scores, and depressive symptom improvement. The models were subsequently retrained without including the study version (V1/V2) as a feature to assess its impact. Model generalizability was assessed by training on passive data from the V1 cohort and testing on the V2 cohort.

To study the impact of depression symptom improvement on adherence, differences in average completion rate and PHQ-9 completion rate between improvement groups were assessed using Mann-Whitney U tests. A robust linear model (RLM) regression was performed with improvement status, controlling for baseline PHQ-9 scores, to predict average completion rate. The RLM was chosen over other regression techniques to handle potential outliers in the data [41].

Cox proportional hazards models were constructed to predict retention, measured as time to last PHQ-9 questionnaire completion, between improvers and nonimprovers, incorporating demographic parameters and average scores from other questionnaires as covariates to evaluate their utility in predicting retention. We chose to study retention for the PHQ-9 rather than globally because completion rates and the frequency with which the questionnaires were sent to participants varied significantly between questionnaires, potentially compromising the reliability and validity of a comprehensive retention measure for survival analysis; the PHQ-9 was chosen as a representative questionnaire given its utility in usual clinical practice. Kaplan-Meier curves were plotted for these models to illustrate assessment retention over time.

### Ethical Considerations

Ethical approval for both parent trials (V1: NCT00540865 and V2: NCT01808976) was granted by the Institutional Review Board of the University of California, San Francisco. Participants provided informed consent that included permission for their data to be shared with other researchers for secondary analyses, in line with the National Institute of Mental Health data-sharing policy (NOT-MH-19-033). Our study used only these deidentified datasets and involved no direct interaction with participants.

## Results

### Outcomes

There were 2201 participants in the dataset. Among them, 1093 were engaged, meaning they completed at least 1 nonbaseline questionnaire, and 1108 were disengaged. In the engaged group, the median average completion rate was 37.6% (IQR 15.5%-67.9%), and the mean was 40.6% (SD 24.2%). Within the engaged group, 548 participants had a mean completion rate higher than the median and were classified as the high completion group (mean 62.3%, SD 11.5%), while 550 participants were classified as the low completion group (mean 19%, SD 9.9%). The average completion rate for the entire cohort was 20.2% (SD 26.5%) for the whole cohort.

### Demographics and Study Parameters

Kruskal-Wallis tests and Mann-Whitney U tests showed significant disparities in average completion rate and engagement between groups defined by gender, education, income satisfaction, last-year income, race, device used, study arm, and study version (Table 1). The findings on average completion rate align with previous literature [16,25,26], while the engagement findings are novel, with engagement defined as whether participants chose to remain in the study after completing the baseline assessments.

**Table 1.** Significant differences (*P*<.05) in engagement rates between groups defined by demographics or study parameters.

Category^a^	Engaged (n=1093)	Disengaged (n=1108)	*P* value^b^
Sex, n (%)			
Male	236 (21.59)	311 (28.07)	.025
Female	857 (78.41)	797 (71.93)	.025
Education, n (%)			
Graduate degree	216 (19.76)	132 (11.91)	2.9×10^-5^
Income, n (%)			
<US $20,000	279 (25.53)	391 (35.29)	3.9×10^-5^
$US 60,000‐US $80,000	277 (25.34)	172 (15.52)	7.1×10^-7^
Income satisfaction, n (%)			
Cannot make ends meet	769 (70.36)	598 (53.97)	1.6×10^-13^
Have enough to get along	194 (17.75)	360 (32.49)	1.2×10^-13^
Race, n (%)			
Asian	95 (8.69)	54 (4.87)	.024
Non-Hispanic White	640 (58.55)	524 (47.29)	7.3×10^-6^
Hispanic or Latino	191 (17.47)	366 (33.03)	3.4×10^-15^
Study arm, n (%)			
iPST^c^	252 (23.06)	77 (6.95)	2.8×10^-24^
Project: EVO	354 (32.39)	181 (16.34)	1.2×10^-16^
HealthTips	369 (33.76)	151 (13.63)	8.7×10^-27^
Study, n (%)			
Brighten-v1	742 (67.89)	376 (33.94)	3.7×10^-55^
Brighten-v2	351 (32.11)	732 (66.06)	3.7×10^-55^

a Percentages for a given category were calculated on the total number of engaged or disengaged participants. A participant is defined as being “engaged” if they completed at least 1 nonbaseline questionnaire of any kind. Participants who dropped out of the study immediately after baseline, without completing any nonbaseline questionnaire, are classified as “disengaged.”

b Bonferroni correction applied.

c iPST: iProblemSolve.

### Symptomatology

To evaluate the impact of baseline symptomatology and average symptom severity over the course of the study on adherence, we compared baseline questionnaire scores and average questionnaire scores between high and low completion groups. After Bonferroni correction, the mean scores of the PHQ-2, PHQ-9, and PGIC questionnaires collected over the 12-week duration of the study were significantly different between high and low completion groups (*P*<.001 for PHQ-2 and PHQ-9 and *P*=.002 for PGIC). Participants with lower PHfQ-2 scores and PHQ-9 scores (ie, less depressed participants), and participants with higher PGIC scores (ie, those who perceived better clinical improvement since the beginning of the study) were more likely to be in the high completion group. This suggests that participants with lower depression levels and perceived improvement in their clinical condition showed greater adherence compared to those with higher depression levels and perceived worsening of their condition (Table 2). Mean baseline PHQ-9 score was 13.9 (SD 4.8) for the engaged group and 14.4 (SD 5.0) for the disengaged group, with the difference being statistically significant (*t*_1917_=−2.53; *P*=.01). This indicates that participants who were less depressed at baseline were more likely to complete at least 1 nonbaseline questionnaire; however, these baseline scores were not predictive of adherence in subsequent assessments.

**Table 2.** Comparisons of questionnaire scores averaged over the 12-week study period between high and low completion groups.

Questionnaire	High completion rate (SD)	Low completion rate (SD)	*t* score (*df*)	*P* value^a^
Mean score				
PHQ-2^b^	4.50 (1.52)	5.20 (1.84)	−6.793 (1019)	1.49×10^-10^
PHQ-9^c^	9.30 (4.87)	11.27 (5.43)	−5.632 (752)	2.01×10^-7^
Sleep assessment	7.23 (1.68)	7.43 (2.09)	−1.625 (768)	.837
SDS^d^	21.32 (8.50)	22.80 (9.21)	−2.658 (968)	.064
PGIC^e^	2.66 (0.97)	2.40 (1.07)	3.78 (744)	.001
Baseline score				
PHQ-9	13.75 (4.83)	13.96 (4.86)	−0.709 (1091)	≥.99
ALC^f^	3.50 (2.61)	3.64 (2.60)	−0.757 (773)	≥.99
GAD-7^g^	11.26 (5.78)	11.87 (5.66)	−1.529 (764)	≥.99

a Bonferroni correction applied.

b PHQ-2: Patient Health Questionnaire-2.

c PHQ-9: Patient Health Questionnaire-9.

d SDS: Sheehan Disability Scale.

e PGIC: Patients Global Impression of Change.

f ALC: Alcohol Use Questionnaire.

g GAD-7: Generalized Anxiety Disorder 7-Item Scale.

### Clinical Improvement

**Figure 1.**
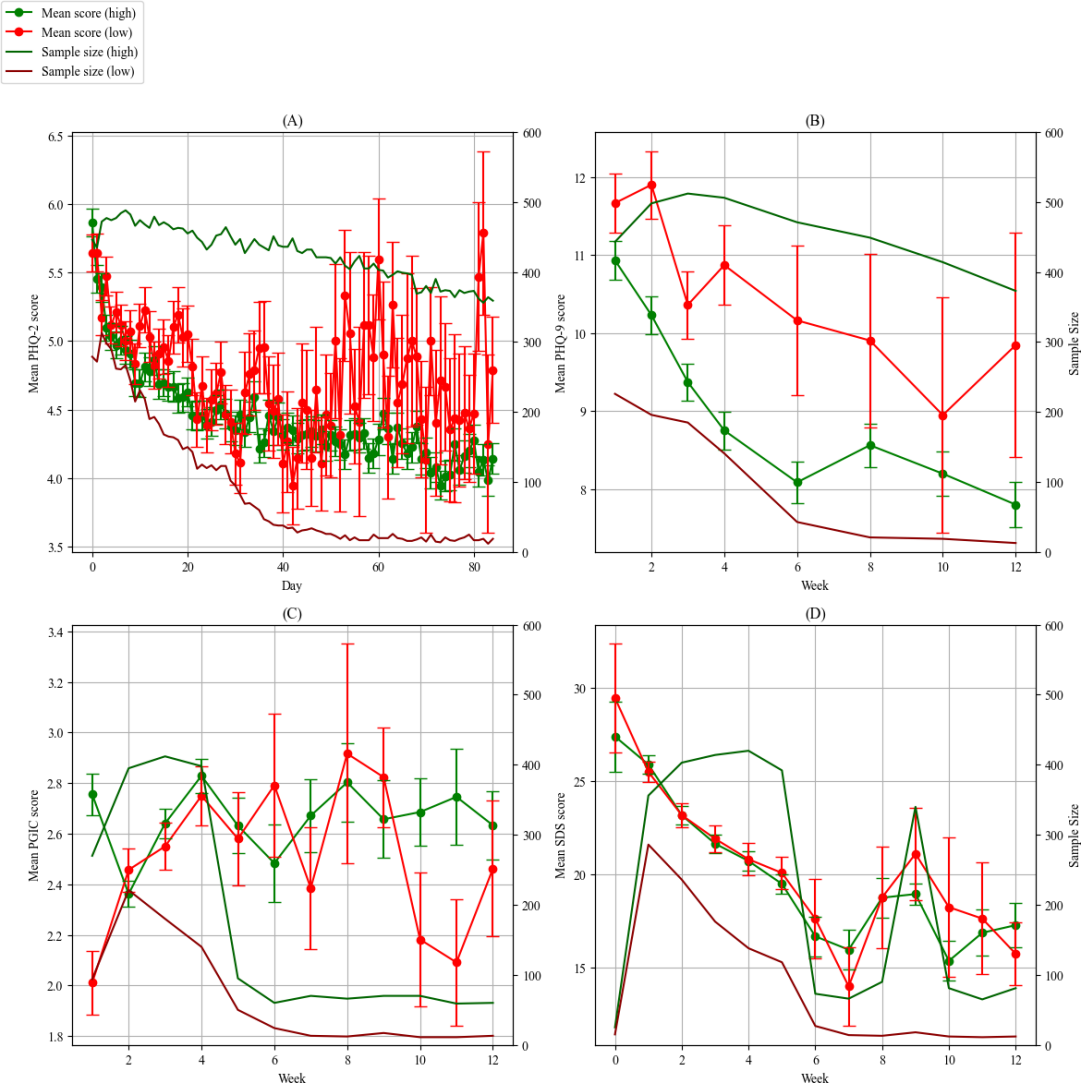
Mean scores over time for high- and low-completion groups by assessment, including sample sizes. (A) Mean Patient Health Questionnaire (PHQ)-2 score over time by completion group, (B) mean PHQ-9 score over time by completion group, (C) mean Patients Global Impression of Change (PGIC) score over time by completion group, and (D) mean Sheehan Disability Scale (SDS) score over time by completion group.

To investigate the longitudinal relationship between questionnaire scores and adherence, we conducted a repeated measures GLM model of mean score over time for both high and low completion groups. We found that there was significant improvement in PHQ-2 scores (coeff=−0.01, 95% CI −0.01 to −0.01; *P*<.001) and PHQ-9 scores (week: coeff=−0.24, 95% CI −0.35 to −0.14; *P*<.001) over time for both high and low completion rate groups. Daily PHQ-2 scores were not significantly different between the 2 groups (coeff=0.04, 95% CI –0.14 to 0.22; *P*=.70), but the effect of time on the scores was statistically significant, with participants from the high completion group having a greater decrease in score over time (coeff=–0.012, 95% CI –0.014 to –0.009; *P*=.001) (Figure 1A). Averages of PHQ-9 score for each weekly (or biweekly) assessment over time were significantly different between the 2 groups (coeff=1.29, 95% CI 0.24 to 2.34; *P*=.021), but the longitudinal trend was similar (coeff=0.03, 95% CI –0.123 to 0.184; *P*=.70), with PHQ-9 scores decreasing significantly over time (coeff=–0.24, 95% CI –0.35 to –0.13; *P*<.001) (Figure 1B). Furthermore, the decrease in sample size regarding completion of PHQ-9 and PHQ-2 was significantly different between high and low completion groups (*P*_PHQ-9_=.02 and *P*_PHQ-2_<.001), with the sample size from the low completion group decreasing more rapidly. For other questionnaires, such as the sleep assessment, PGIC, and SDS, there was no statistically significant difference in the rate of sample size decrease between the high and low completion groups. There were no significant differences in SDS, PGIC, and sleep assessment scores between the 2 groups, although SDS scores decreased significantly over time for the 2 groups in a similar fashion (coeff=–0.84, 95% CI –1.17 to –0.50; *P*<.001). The effect of time on sleep assessment scores was found to be different between the groups, but it was nonsignificant (*P*=.08; Figure 1).

**Figure 2.**
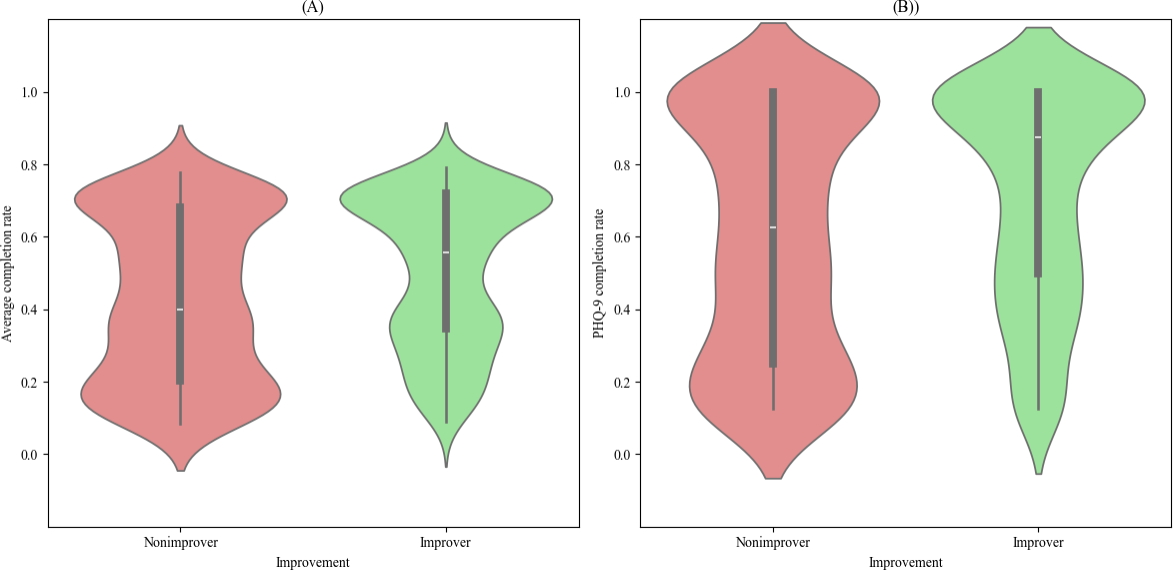
Comparison of adherence between improvers and nonimprovers. (A) Average completion rate (Mann-Whitney U score=127084; *P*<.0001 and (B) Patient Health Questionnaire (PHQ)-9 completion rate (Mann-Whitney U score=125418; *P*<.0001). Average completion rate is defined as the total number of questionnaires completed divided by the total number of questionnaires sent to participants. PHQ-9 completion rate is defined as the total number of PHQ-9 questionnaires completed divided by the total number of PHQ-9 questionnaires sent to participants.

To evaluate the association between clinical improvement regarding depressive symptoms and adherence, we divided the engaged cohort into improvers and nonimprovers. The categorization was based on the difference between participants’ baseline PHQ-9 score and their latest PHQ-9 score, using the minimal clinically important difference of 5 points as the threshold for meaningful improvement [39]. There were 410 “improvers” and 513 “nonimprovers.” Between those groups, there was a significant difference in average completion rate (improvers: mean 50.7%, SD 20.6%; median 55.5%; nonimprovers: mean 42.6%, SD 22.7%; median 39.9%; and Mann-Whitney U=127,084; *P*<.001), PHQ-9 completion rate, and baseline PHQ-9 score (*P*<.001 for all comparisons), with “improvers” having higher baseline PHQ-9 scores than “nonimprovers” (Mann-Whitney U=137,574; *P*<.001) (Figure 2A and 2B). Mean baseline PHQ-9 score was 15.3 (SD 4.7) for improvers and 12.6 (SD 4.6) for nonimprovers. The RLM regression model showed that the average completion rate of “nonimprovers” was, on average, 8.85% (*P*<.001) lower than that of “improvers.” However, for both groups, baseline PHQ-9 scores were not significantly associated with average completion rates (*P*=.06; Figure 2).

**Figure 3.**
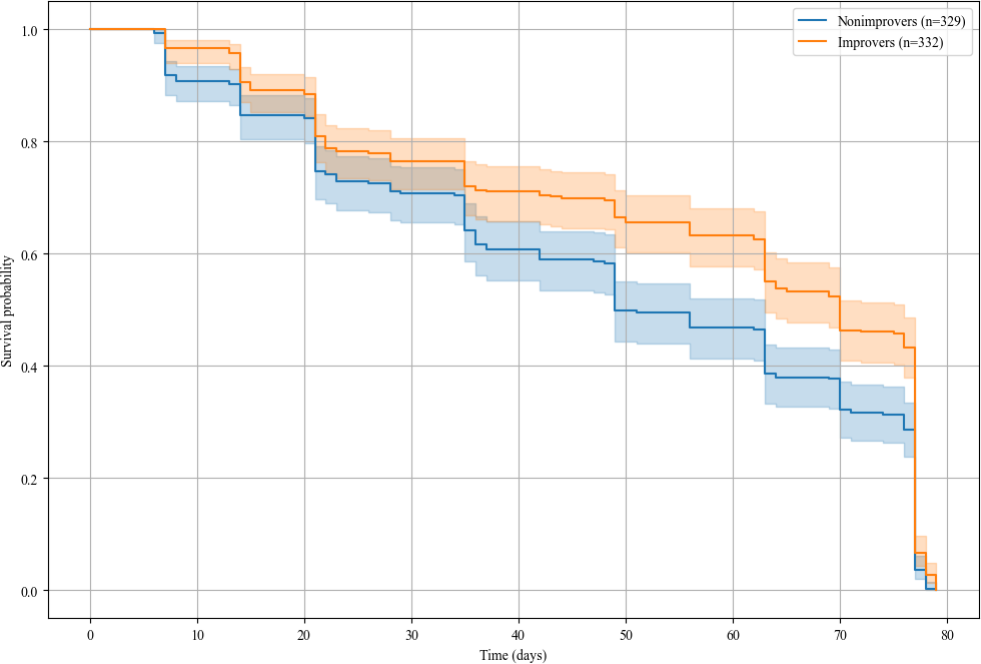
Kaplan-Meier curves showing retention for improvers versus nonimprovers, with the event defined as the last Patient Health Questionnaire (PHQ)-9 questionnaire completed within the 12-week study duration (n=661). Significant survival differences were observed (improver Coef=−0.25, Exp(coef)=0.78; *P*<.005). Key predictors: age: Coef=−0.01, Exp(coef)=0.99; *P*<.005); mean score sleep: Coef=0.06, Exp(coef)=1.06; *P*=.01); and study Brighten-v2: Coef=0.79, Exp(coef)=2.19; *P*<.005). Bonferroni correction was applied.

To study PHQ-9 retention, defined as the duration for which participants continued completing the PHQ-9, we performed a Cox proportional hazards model to compare improvement groups, using demographic parameters [1], average scores from other questionnaires, and study version as covariates. The Cox proportional hazards model revealed significant differences in survival probabilities between the 2 groups (hazard ratio 0.78, 95% CI 0.67-0.91; *P*=.002), with the event defined as the day a participant completed their last PHQ-9 questionnaire within 12 weeks from the start of the study. The median survival time was 70 days (mean 56.8, SD 24.7) for improvers (n=332) and 49 days (mean 49.4, SD 25.6) for nonimprovers (n=329), indicating that nonimprovers stopped completing PHQ-9 questionnaires 7.4 days earlier on average compared to improvers (Mann-Whitney U=64632; *P*<.001). We found that age (*P*=.002), mean score of the sleep assessment (*P*=.01), and study version (*P*<.001) were significant predictors of PHQ-9 retention (Figure 3). Cox proportional hazards model based on the PHQ-2 questionnaire is provided in Multimedia Appendix 2.

### Key Indicators of Adherence and Engagement

**Figure 4.**
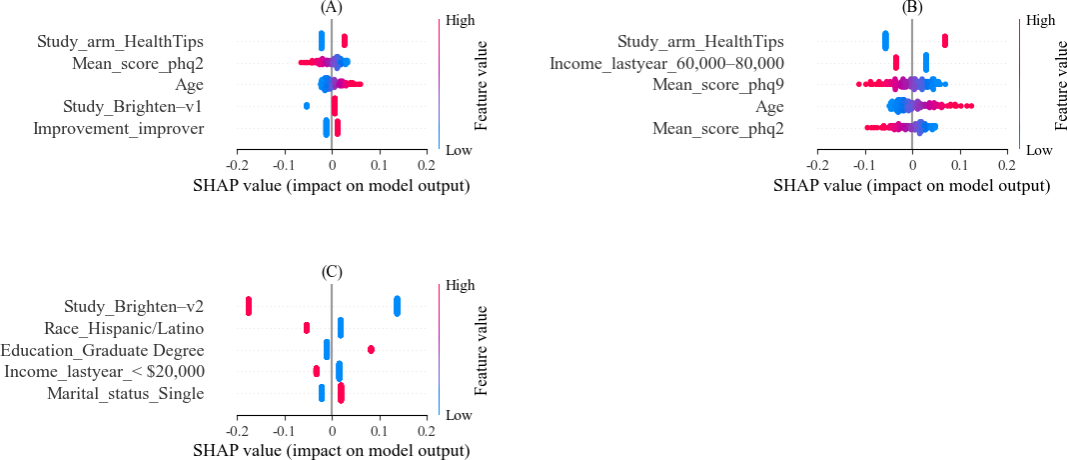
Top 5 features and their Shapley additive explanations (SHAP) value for predicting compliance metrics using elastic net regression. (A) Top 5 features for predicting average completion rate (n=701; mean squared error [MSE]=0.04), (B) top 5 features for categorizing participants into high and low completion groups (n=701; *F*_1_-score=0.72), and (C) top 5 features for predicting whether a participant will be “engaged” or “disengaged” (n=2201; *F*_1_-score accuracy=0.66).

To assess what features were most statistically useful in predicting average completion rate and engagement, we trained an elastic net regression model on 80% of the data and tested it on the remaining 20%. SHAP values were used to assess the relative importance of each feature and evaluate their contributions to the model’s predictions (Figure 4). For predicting average completion rate, the 5 most useful features, in order of importance, were (n=701; mean squared error [MSE]=0.04) (1) being in the control group, (2) having a low mean PHQ-2 score, (3) being older, (4) being in the V1 cohort, and (5) improving in depressive symptoms (Figure 4A).

The 5 most important features associated with being in the high completion group were (n=701; *F*_1_-score=0.72) (1) being in the control group, (2) not being in the US $60,000-US $80,000 annual income group, (3) having a low mean PHQ-9 score, (4) being older, and (5) having a low mean PHQ-2 score (Figure 4B).

The 5 most important features associated with “engaging” in the study were (n=2201; *F*_1_-score=0.65) (1) being in the V1 cohort, (2) not being a Hispanic or Latino person, (3) having a graduate degree, (4) having an annual income greater than US $20,000, and (5) being single (Figure 4C).

To assess the impact of study version (V1/V2) on the models’ predictability, we conducted an exploratory analysis excluding it from the features and found that the categorization models (engagement and completion group) performed slightly worse, although the 5 most important features were generally similar (*F*_1_-score decreased from 0.72 to 0.67 for completion group categorization and from 0.66 to 0.60 for engagement categorization). The average completion rate model MSE did not change, although the *R*^2^ decreased from 0.16 to 0.13.

When trained on the V1 cohort and tested on the V2 cohort, the categorization models performed poorly (*F*_1_-score=0.32 for engagement categorization and *F*_1_-score=0.46 for completion group categorization). The model predicting average completion rate had slightly worse predictive performance (MSE=0.04; *R*^2^=0.12).

## Discussion

### Principal Findings

We found that the subjective perception of a participant’s own clinical trajectory (PGIC score) was associated with assessment adherence, with participants who reported a perceived improvement in their clinical condition showing greater adherence. We also found that increased depressive symptoms at baseline were not significantly associated with adherence, but rather with engagement, thus suggesting that more depressed participants were more likely to withdraw immediately after baseline assessment. Furthermore, participants who improved regarding depressive symptoms had greater assessment adherence and retention, despite having higher depressive symptoms at baseline. Previous research has established a link between baseline depressive symptoms and engagement [13]. This study makes novel contributions by exploring the associations between perceived clinical improvement and adherence, as well as the connection between depressive symptom improvement, baseline depression severity, retention, and adherence.

Despite offering monetary incentives, the researchers conducting the Brighten study observed poor engagement and adherence, highlighting the need to investigate this issue further and explore innovative strategies to address this limitation [25]. The demographic parameters we found associated with lower adherence and engagement are similar to what has been found in previous literature (ie, male sex, lower education, lower income, poorer income satisfaction, and Hispanic or Latino race were associated with poorer adherence) [16,25,26]. However, we found that a perceived worsening of clinical condition throughout the study was associated with less assessment adherence, suggesting that a participant’s own perception of their change in activity, symptoms, emotion, and quality of life can influence their willingness to complete assessments.

Regarding completion of the PHQ-9 questionnaire, there was a significant difference in retention probability, with participants who improved in depressive symptoms having more chance of continuing to complete assessments than nonimprovers. Age, mean score of sleep assessment, and study version were significant features for predicting retention. Therefore, the global change in depressive symptoms over time was associated with study retention and adherence rate. These findings highlight the possibility of attrition bias in digital health studies, leading to an overestimation of symptom severity improvement. Furthermore, this kind of bias could lead to misleading conclusions about the effectiveness of treatments and affect clinical decision-making, reinforcing the importance of controlling for attrition rates in digital health studies [42]. However, a possible limitation of the retention analyses is the lack of insight regarding improvement for nonimprovers who stopped completing answering questionnaires early in the study, as they may have eventually experienced symptom improvement that went unrecorded.

The study arm was the most important indicator of assessment completion rate, with participants from the control group having higher completion rates than participants using the Project: EVO app or the iPST app. Moreover, the finding that participants in the iPST group, who were encouraged to use the app once per week, showed higher adherence than those in the Project: EVO group, who were asked to use it 6 times per week, suggests a trade-off between effort and study engagement. Participants required to invest more time in the digital intervention were more likely to skip assessments. We found that baseline PHQ-9 score, although predictive of engagement (serving as an initial “filter” for participants continuing in the study), was not an important indicator of adherence, highlighting the need to focus on clinical trajectories in addition to static measures. In addition, participants who improved significantly in their depressive symptoms had higher completion rates than participants who did not, and their baseline PHQ-9 scores were significantly higher, although, as mentioned, baseline PHQ-9 score is not a direct indicator of completion rate. This suggests that while initial severity is not directly linked to longitudinal adherence, positive clinical progress may encourage consistent assessment completion. In fact, we found that participants who significantly improve regarding depressive symptoms are less likely to withdraw from active completion of assessments than participants who do not. Interestingly, it is also worth considering that the capacity to regularly complete questionnaires might be a potential predictor of future improvement in depressive symptoms.

### Limitations

This study presents several limitations. The most important limitation is the lack of data on participants’ use of the intervention apps, particularly the project: evo group, whose participants were encouraged to use the app 6 times per week. As a result, we were unable to assess whether engagement with the intervention apps influenced participants’ engagement with questionnaire completion. However, our primary interest was in questionnaire completion, as this measure is more directly comparable across digital health studies and thus is the most applicable for broader research contexts. In addition, the GLM repeated measures analysis revealed intriguing patterns and trends in the effect of time and adherence on questionnaire scores, but the rapid decline in sample size over time limits the strength of any conclusions that can be drawn. The elastic net regression models showed only modest performance, with clear potential for improvement. Feature engineering, the collection of larger feature sets, hyperparameter tuning, and time-series transformations are potential strategies that could enhance the accuracy and robustness of the models by improving their ability to recognize underlying patterns, as well as identify temporal dynamics within the data [43-45]. Given the model’s limited fit, SHAP value interpretations for top features should be regarded as exploratory. Furthermore, we were not able to generalize the model’s predictability across cohorts, highlighting the need for further validation with diverse datasets to ensure its applicability in a realistic transdiagnostic population [46,47]. The limited data available for passively collected variables, such as communication and mobility features, resulted in inconclusive analyses including these variables (refer to Multimedia Appendix 3).

### Future Directions

To address adherence and retention challenges in future studies, implementing continuous monitoring systems to identify participants at risk of dropping out could be beneficial [48]. Targeted outreach strategies, such as personalized reminders or support interventions, may help engage these individuals and improve adherence [49,50]. Monetary incentives have also been shown to be effective in increasing adherence and retention rates, though this may be impractical when attempting more naturalistic designs [51-53]. Beyond these approaches, qualitative research is crucial to better understand the underlying reasons for disengagement. Previous user-centered studies indicate that underserved users often cite stigma, cultural mismatch, and usability issues as barriers, while individuals with lower education or digital literacy report practical challenges and a higher perceived time burden when using mobile health apps [54,55]. Conducting more extensive qualitative work would provide valuable insights into how participants perceive their improvement and clinical trajectories, thereby offering a more comprehensive understanding of user challenges and informing strategies to strengthen engagement, adherence, and retention.

### Conclusion

Although demographics are useful in predicting adherence, engagement, and retention in digital health studies, we show that dynamic, measurable parameters regarding symptomatology and symptom change are also important in understanding longitudinal patterns of attrition and adherence, and that they play a crucial role in shaping the remaining cohort as long-term studies progress over time [16,24]. We have shown that participants’ impressions of their own improvement and the clinical trajectory of participants regarding depressive symptoms are important indicators of engagement, adherence, and retention. Further research is needed to assess the causality and implications of this effect and to determine optimal strategies for countering the bias that this phenomenon introduces into datasets.

## Supplementary material

10.2196/69464 Multimedia Appendix 1 Significant differences in average completion rate between groups defined by demographics or study parameters, and questionnaires analyzed in the study.

10.2196/69464 Multimedia Appendix 2 Cox proportional hazards model for improvers and nonimprovers based on Patient Health Questionnaire (PHQ)-2 scores, with the event defined as the last PHQ-2 questionnaire completed within the 12-week study duration.

10.2196/69464 Multimedia Appendix 3 Top 5 features and their Shapley additive explanations (SHAP) values for predicting adherence metrics using elastic net regression trained on demographics, study parameters, average questionnaire scores, and passive data features.

## Abbreviations

GLM: generalized linear model
iPST: iProblemSolve
MSE: mean squared error
PGIC: Patients Global Impression of Change
PHQ-2: Patient Health Questionnaire-2
PHQ-9: Patient Health Questionnaire-9
RLM: robust linear model
SDS: Sheehan Disability Scale
SHAP: Shapley additive explanations
