# Supplementary material for: Dynamic Indicators of Adherence and Retention in Adults Using a Digital Mental Health App: Longitudinal Observational Analysis From the Brighten Study
Source: JMIR Hum Factors. 2025 Dec 22;12:e69464. doi: 10.2196/69464 (PMC12721583; doi:10.2196/69464)
Supplement: Multimedia Appendix 1 [file humanfactors-v12-e69464-s001.docx]

## Multimedia Appendix 1

Significant differences in average completion rate between groups defined by demographics or study parameters.^a,b,c^

| **Category** | **Comparison** | **u-statistic** | **H-statistic** | ***P* value** |
| --- | --- | --- | --- | --- |
| Gender | Female vs Male | 419267 |  | 5.94E-03 |
| Education | Graduate Degree vs High School |  | 41.13751677 | 6.11E-08 |
|  | Graduate Degree vs University |  |  | 4.14E-05 |
|  | Graduate Degree vs Community College |  |  | 2.00E-06 |
| Income satisfaction | Can't make ends meet vs Am comfortable |  | 107.2762363 | 4.15E-04 |
|  | Can't make ends meet vs Have enough to get along |  |  | 4.12E-24 |
|  | Am comfortable vs Have enough to get along |  |  | 8.79E-04 |
| Last year income ($) | 60,000-80,000 vs 20,000-40,000 |  | 69.50172645 | 9.55E-05 |
|  | 60,000-80,000 vs < 20,000 |  |  | 3.41E-11 |
|  | 60,000-80,000 vs 100,000+ |  |  | 4.60E-07 |
|  | 80,000-100,000 vs < 20,000 |  |  | 4.68E-04 |
|  | 80,000-100,000 vs 100,000+ |  |  | 5.24E-04 |
|  | 40,000-60,000 vs < $20,000 |  |  | 2.58E-03 |
| Race | Asian vs Hispanic/Latino |  | 100.8484504 | 1.01E-09 |
|  | African-American/Black vs Hispanic/Latino |  |  | 2.67E-04 |
|  | Non-Hispanic White vs Hispanic/Latino |  |  | 7.97E-18 |
|  | Hispanic/Latino vs More than one |  |  | 3.07E-05 |
| Heard about us | others vs Twitter/Facebook |  | 27.2057934 | 4.49E-04 |
| Device | iPhone_vs_Android | 429311.5 |  | 1.43E-04 |
| Study arm | iPST vs EVO |  | 21.77318875 | 1.26E-02 |
|  | EVO vs HealthTips |  |  | 1.53E-05 |
| Study | Brighten-v1_vs_Brighten-v2 | 863586 |  | 8.29E-77 |

^a^Average completion rate is defined as the total number of questionnaires completed divided by the total number of questionnaires that were sent to participants.

^b^Bonferroni correction applied for >2 comparisons (Kruskal-Wallis tests)

To investigate the effect of baseline depressive symptoms on adherence, an Ordinary Least Squares (OLS) model on baseline PHQ-9 score and average completion rate was performed. We found no significant association between baseline PHQ-9 score and average completion rate (R^2^=0.001, p=0.4), suggesting that adherence is affected in a longitudinal way rather than by baseline metrics.

^c^Demographics items were:

- Gender (male or female)
- Age (integer)
- Education (elementary school, high school, community college, university, graduate degree)
- Income satisfaction (“can’t make ends meet”, “have enough to get along”, “am comfortable”) Last year income (in American dollars; < 20,000; 20,000-40,000; 40,000-60,000; 60,000-80,000; 80,000-100,000; >100,000)
- Race (Asian, African American/Black, Non-Hispanic White, Hispanic/Latino, American Indian/Alaskan Native, Native Hawaiian/other Pacific Islander, More than one, Other)
- Working status (yes or no)
- Marital status (single, married/partner, separated/widowed/divorced)
- How they heard about the study (Craigslist, advertisement, friend/colleague, through other studies, Twitter/Facebook, other)
- Device used (iPhone, Android)

Questionnaires

**Patient Health Questionnaire (PHQ)-9**

**
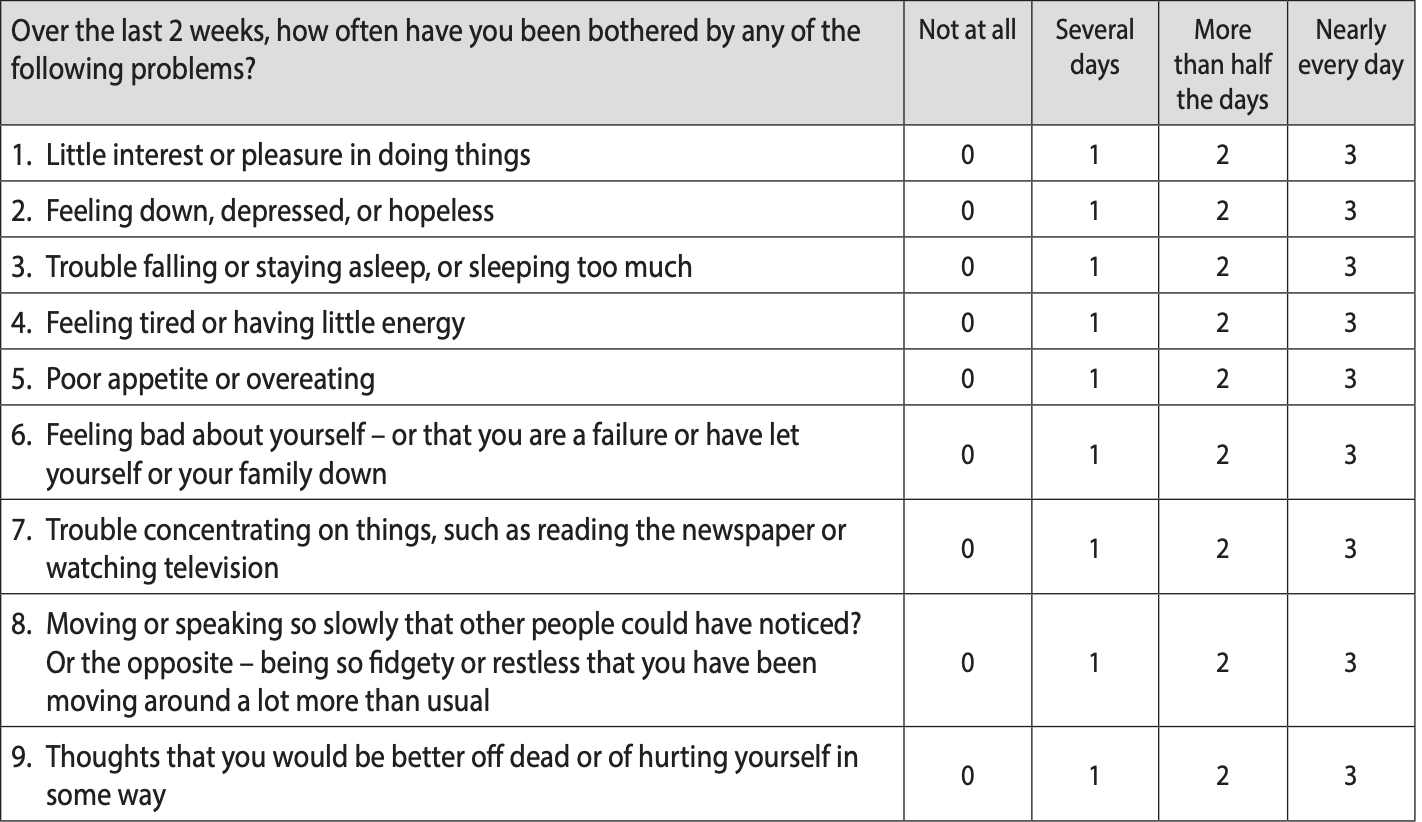
**

**Patient Health Questionnaire (PHQ)-2**


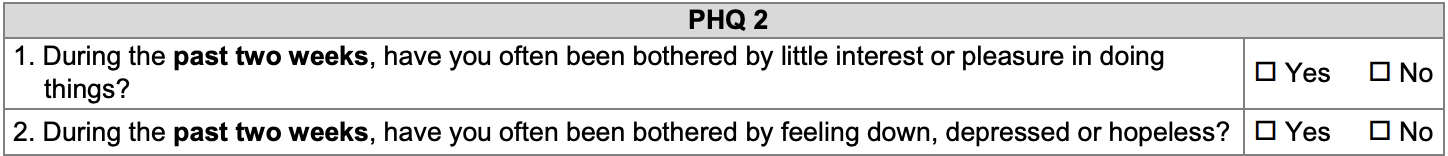


**3-Item Sleep Assessment**

**Example: Thinking about your time in bed at night, over the past week, on average how long…**

- 1. **Did it take you to fall asleep?**

**Patients’ Global Impression of Change Scale (PGIC)**


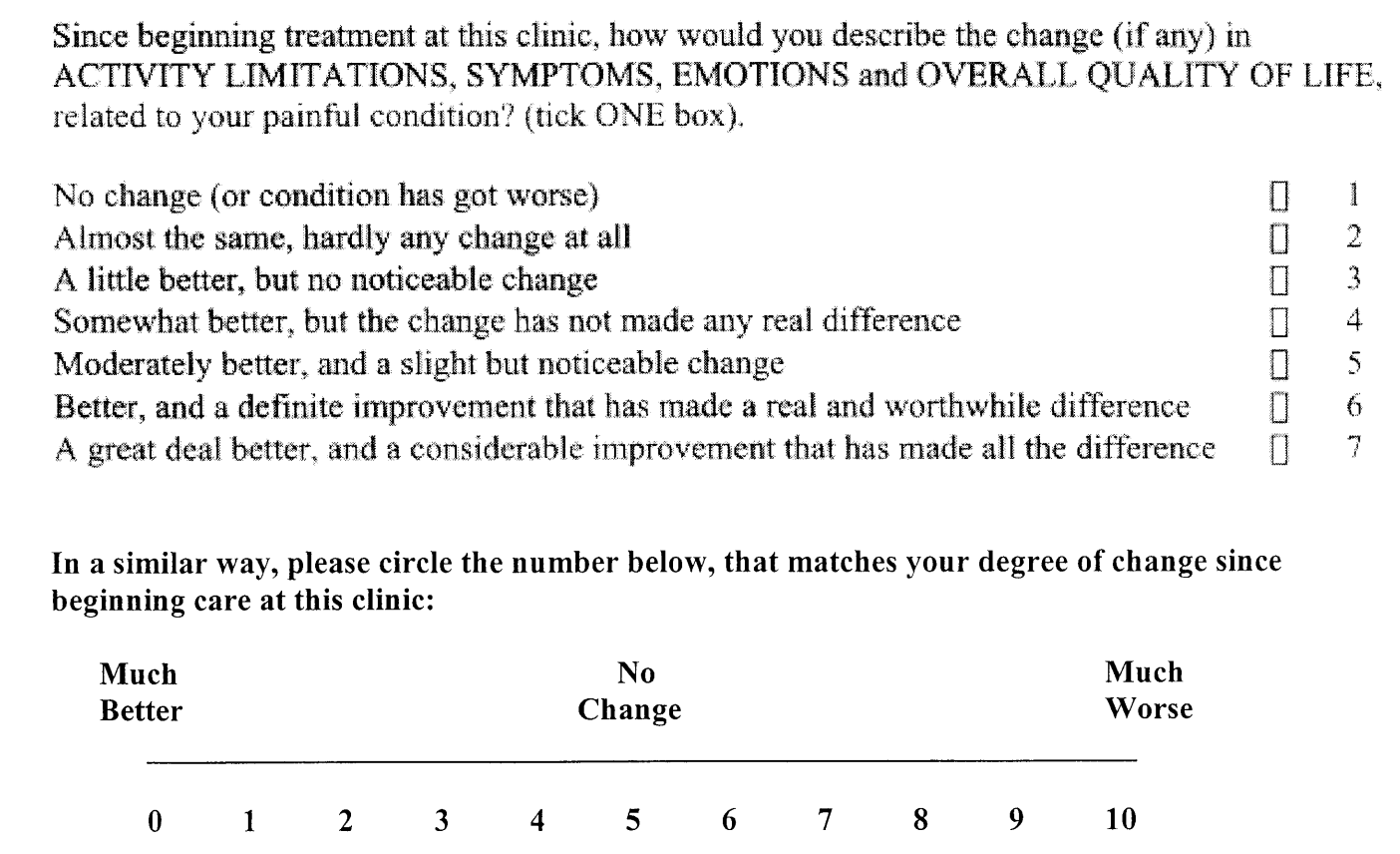


**Sheehan Disability Scale (SDS)**

Example of item: “The symptoms have disrupted your work / schoolwork:” (circle item from 0 to 10)

0: not at all

1-3: mildly

4-6: moderately

7-9: markedly

10: extremely

**Mental Health Services Use Survey**

**Examples:**

1. **Do you see a psychiatrist for mental health or substance abuse treatment?**
2. **Do you see a psychologist, social worker, or other counselor for mental health or substance abuse treatment?**

**Study App Satisfaction Survey (4 items)** including their expectations, overall satisfaction, effectiveness in meeting needs, and usability ratings.

**Alcohol Use Questionnaire** (answers are integers; there are 3 items)

Example

1. How often did you have a drink containing alcohol in the past year?

**Other Health-Related Apps Use Survey**

Assessing use, in past week, of any health-related apps (alcohol, concentration, exercise and fitness, medical, mood, pain management, or relaxation)
