# Supplementary material for: Dynamic Indicators of Adherence and Retention in Adults Using a Digital Mental Health App: Longitudinal Observational Analysis From the Brighten Study
Source: JMIR Hum Factors. 2025 Dec 22;12:e69464. doi: 10.2196/69464 (PMC12721583; doi:10.2196/69464)
Supplement: Multimedia Appendix 2 [file humanfactors-v12-e69464-s002.docx]

## Multimedia Appendix 3

Cox PH model regarding PHQ-2 assessments was performed. Improvement was determined by subtracting the most recent score by the oldest score, considering any difference as clinically significant (MCID=0). When predicting retention for PHQ-2 completion, the following features were significant: age (*P*<.005), Hispanic/Latino (*P*=.01) race (*P*<.005), study version (*P*<.005), being in the control group (*P*<.005; Figure 5).


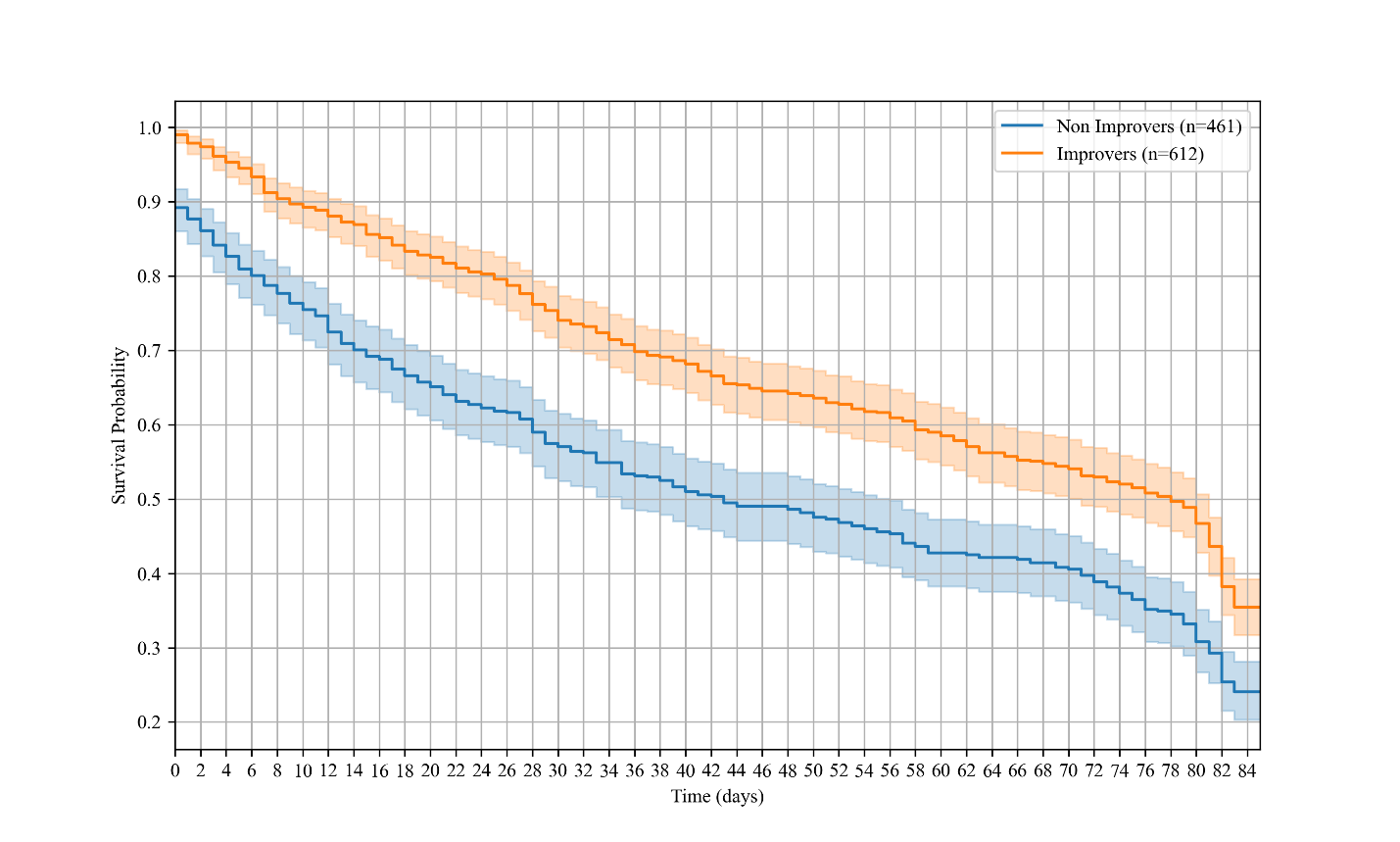


**Figure 5.** Kaplan-Meier curve showing retention of improvers versus nonimprovers based on PHQ-2 scores, with the event defined as the last PHQ-2 questionnaire completed within the 12-week study duration (n=1073). Significant survival differences noted (Improver Coef=-0.42, Exp(coef)=0.66; *P*<.005). Key predictors: Age: Coef=-0.02, Exp(coef)=0.98; *P*<.005). Race Hispanic/Latino: Coef=0.23, Exp(coef)=1.26; *P*=.01). Study Brighten-v2: Coef=0.83, Exp(coef)=2.29; *P*<.005. Study arm Health Tips (control group): Coef=-0.41, Exp(coef)=0.66; *P*<.005. Bonferroni correction applied.
