# Supplementary material for: Dynamic Indicators of Adherence and Retention in Adults Using a Digital Mental Health App: Longitudinal Observational Analysis From the Brighten Study
Source: JMIR Hum Factors. 2025 Dec 22;12:e69464. doi: 10.2196/69464 (PMC12721583; doi:10.2196/69464)
Supplement: Multimedia Appendix 3 [file humanfactors-v12-e69464-s003.docx]

## Multimedia Appendix 2

Passive features were used as features, in addition to demographics, study parameters, and average questionnaire scores to see if they would help the Elastic Net Regression models predict average completion rate and completion group (high vs low). Four models were built, two for V1 
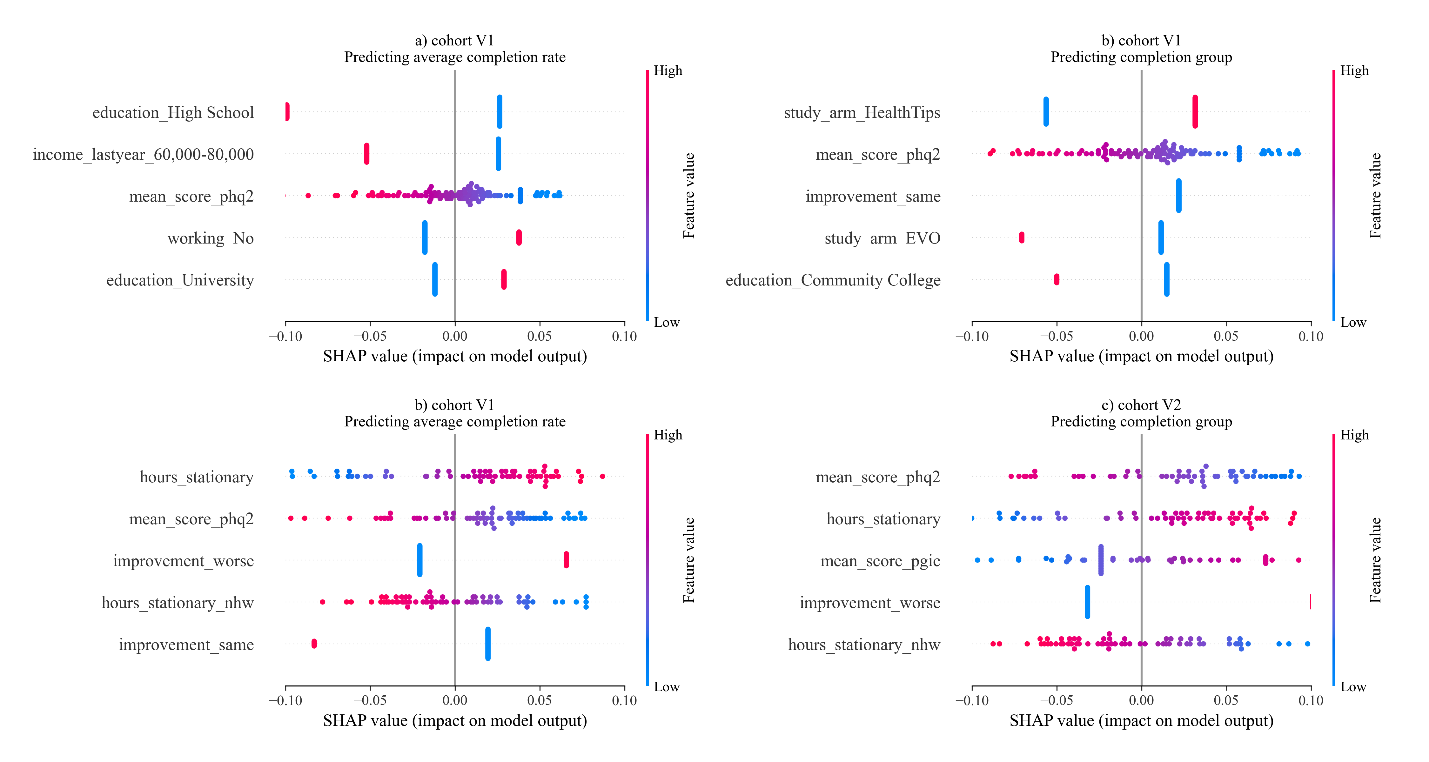
and two for V2, as the features measured were different between the two cohorts. Only mobility features were used to train the V2 models, because there was not enough data to work with features related to communication. Overall, models trained with passive feature data did not show improved performance. However, high attrition rates and low engagement with passive data collection limit our ability to conclude whether passive features could be associated with compliance in digital health studies (Figure 6).

**Figure 6:** Top 5 features and their SHAP value for predicting adherence metrics using Elastic Net Regression trained on demographics, study parameters, average questionnaire scores, and passive data features. Passive features for V1 were: aggregate_communication, call_count, call_duration, interaction_diversity, missed_interactions, mobility, mobility_radius, sms_count, sms_length, unreturned_calls. Passive features for V2 were: came_to_work, distance_active, distance_high_speed_transportation, distance_powered_vehicle, distance_walking, hours_accounted_for, hours_active, hours_high_speed_transportation, hours_of_sleep, hours_powered_vehicle, hours_stationary, hours_stationary_nhw, hours_walking, location_variance. (A) Top 5 features for predicting average completion rate in the V1 cohort (n=112; MSE=0.05), (B) top 5 features for categorizing patients into high and low completion groups in the V1 cohort. (n=112; *F*_1_-score accuracy=0.70), (C) top 5 features for predicting average completion rate in the V2 cohort (n=67; MSE=0.04), (D) top 5 features for categorizing patients into high and low completion groups in the V2 cohort. (n=67; *F*_1_-score accuracy=0.63).
